# Prognostic value and clinicopathological features of PD-1/PD-L1 expression with mismatch repair status and desmoplastic stroma in Chinese patients with pancreatic cancer

**DOI:** 10.18632/oncotarget.14069

**Published:** 2016-12-21

**Authors:** Yu Wang, Jiacheng Lin, Jiujie Cui, Ting Han, Feng Jiao, Zhuo Meng, Liwei Wang

**Affiliations:** ^1^ Department of Medical Oncology and Pancreatic Cancer Center, Renji Hospital, School of Medicine, Shanghai Jiao Tong University, Shanghai 200000, China; ^2^ Department of Medical Oncology and Pancreatic Cancer Center, Shanghai General Hospital, School of Medicine, Shanghai Jiao Tong University, Shanghai 201620, China; ^3^ Shanghai Key Laboratory of Pancreatic Disease, Shanghai 201620, China; ^4^ State Key Laboratory of Oncogene and Related Genes, Shanghai Cancer Institute, Renji Hospital, Shanghai Jiao Tong University School of Medicine, Shanghai 200032, China

**Keywords:** PD-1/PD-L1, mismatch repair enzymes, pancreatic cancer, prognosis

## Abstract

Pancreatic cancer (PC) is a highly lethal cancer. Thus, the immune molecular markers which help to select PC patients are especially important. In this study, we aimed at systematically analyzing the expression of MLH1, MSH2, PD-L1 and PD-1, investigate their clinical significance and prognostic value. We found that high expression of PD-L1 on cancer cell membranes correlated with lymph node metastasis (*P* = 0.033) and strongly correlated with poor-differentiation (*P* = 0.008); high expression of PD-1 on cell membranes of T-cells correlated with well-differentiation (*P* = 0.018) and strongly correlated with advanced T stage (*P* = 0.004); high PD-1 expression was associated with a significantly superior OS and was an independent prognostic factor (*P* = 0.031). Then we found an inverse correlation between MSH2 expression and PD-L1 expression (Spearman correlation coefficient r = −0.295, *P* = 0.004). In subgroup analyses, we observed that PD-1 expression level was associated with OS only at low PD-L1 expression subgroup (*P* = 0.021). Finally, when we stratified the cases into four subgroups based on PD-1 expression and stroma density, we found that patients with high PD-1 expression and dense stroma had a better OS, while patients with low PD-1 expression and moderate stroma showed a worst outcome. Our result may provide more effective molecular markers for immunotherapeutic strategies of PC patients in clinical practice.

## INTRODUCTION

Pancreatic cancer (PC) is a highly lethal cancer, with a 5-year survival of approximately 5% [1–2]. The potentially resectable of PC is less than 20%, for the diagnosis of pancreatic cancer is often difficult, and most patients are diagnosed at an advanced stage. However, even in rescetable cases, the median survival time is only 15–18 months. Recently, surgery, radiotherapy and chemotherapy for pancreatic cancer have been improved, but the effect was limited. Thus, it is very important to find more effective molecular markers for the treatment and prognosis of PC patients in clinical practice.

Program death ligand 1 (PD-L1) is a member of the B7 family of immune-regulatory cell-surface proteins. As a receptor of PD-L1, programmed death-1 (PD-1) is typically expressed on activated T cells. PD-1/PD-L1 axis plays an important role in the negative regulation of cell-mediated immune responses as an immune checkpoint. The expression of PD-1/PD-L1 is up-regulated in many tumors and their microenvironment [3–5]. PC is characterised by an abundant desmoplastic stroma which contains dense activated stellate cells. PC is considered as a non-immunogenic tumor with few effector T cells infiltrating into the tumor tissue. The stroma of PC is poor-vascularized and often prevents T-cell from infiltrating [6–9]. Although the antibodies of PD-1/PD-L1 have shown improving outcome in patients with melanoma, renal cell carcinoma, non-small lung cancer, the results of treating pancreatic cancer with single-agent immune checkpoint inhibitors have been disappointing [10–13]. However, anti-tumor activity was preliminarily reported for some PC patients at the 2014 ASCO annual meeting. Thus, the immune molecular markers which help to select PC patients are especially important [14].

High PD-L1 expression was firstly suggested to predict the response to anti-PD-1/PD-L1 antibody therapies. Then, pembrolizumab has been reported to be highly effective in gastrointestinal cancers in patients with mismatch repair deficiencies [15–16]. Deficiencies of mismatch repair enzymes (MLH1, MSH2, MSH6, and PMS2) were originally identified by Dr. Henry Lynch [17]. Meanwhile, mismatch repair deficiency (MMR-D) cancer appears to be more antigenic than mismatch repair proficient (MMR-P) cancer and has a special susceptibility to immunotherapy. Since somatic mutations have the potential to create immunogenic neoantigens, patients with mismatch repair defects, which promote somatic mutations, may have increased intratumoral effector T cell responses to the neoantigens [15–16]. Thus, effective immune-based therapeutic strategies are highly demanded for the majority of malignancies that are naturally immune quiescent.

Although many studies showed that the expression of PD-1 and PD-L1 was correlated with the clinical outcome in several malignancies including PC [18–19], some studies did not find any prognostic impact of PD-1/PD-L1[20]. Also, the factors which affected the expression of PD-1/PD-L1 in PC were not fully illustrated. Moreover, the role of mismatch repair enzymes in this disease and correlation with PD-1/PD-L1 expression remains largely unknown. In this study, we firstly aim to evaluate the correlation of clinical/pathological characteristics and the expression of PD-1/PD-L1, MLH1, MSH2 respectively and demonstrated the prognostic role of them in a tissue microarray (TMA) including 94 well-documented, clinically annotated PC specimens. Then, we evaluated the correlation of PD-1, PD-L1, MLH1, MSH2 and desmoplastic stroma density and investigated their clinical significance in PC progression.

## RESULTS

### Expression of PD-1, PD-L1, MLH1 and MSH2 in PC tissues and staining characteristics

**Figure 1:**
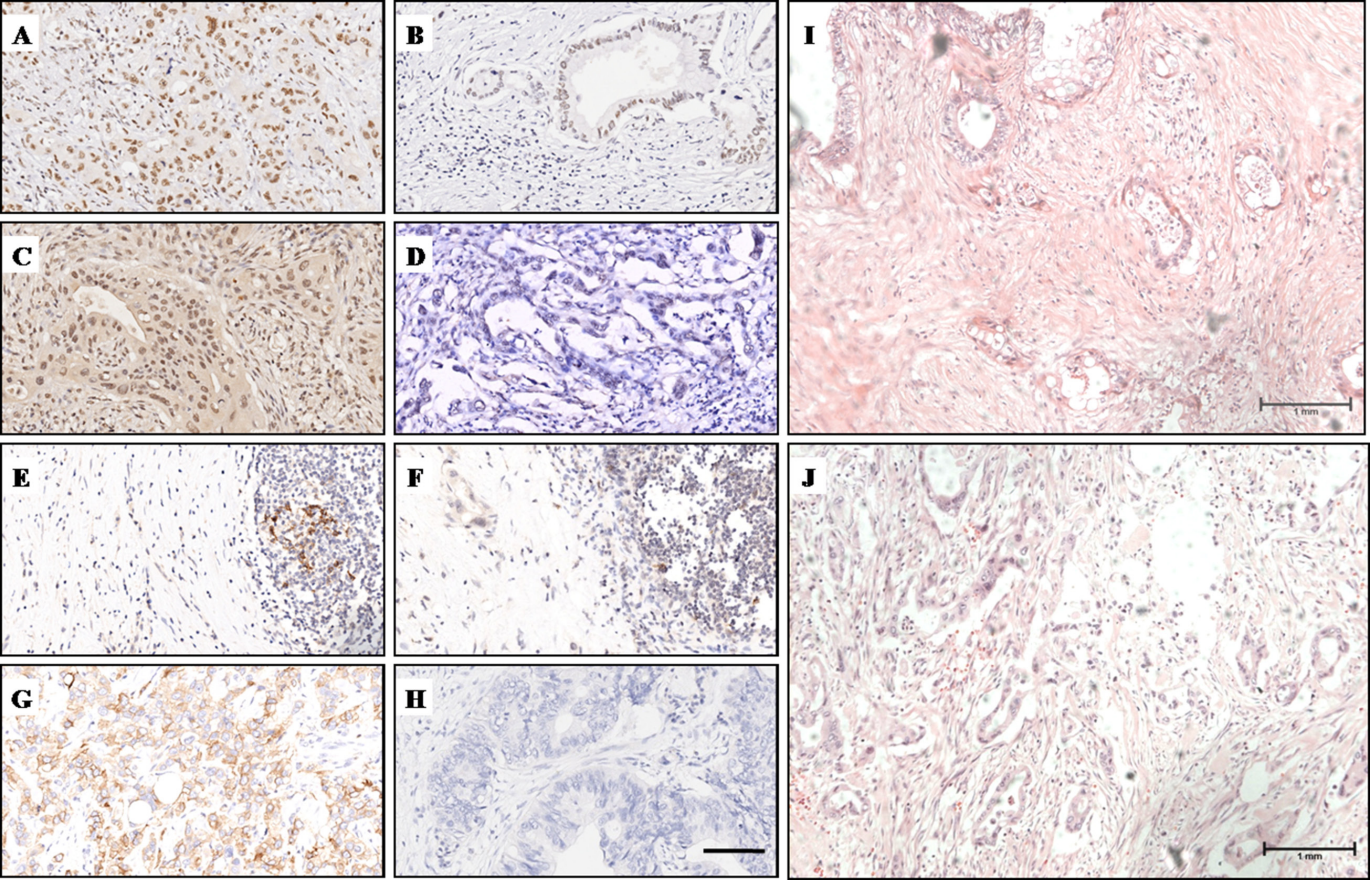
IHC and HE staining characteristics of MLH1, MSH2, PD-1, PD-L1 and stroma density (**A**) High MLH1 expression was observed in the nucleus. (**B**) Low MLH1 expression. (**C**) High MSH2 expression was observed in the nucleus. (**D**) Low MSH2 expression. (**E**) PD-1 was located on cell menbranes of TILs, clusters of PD-1+ TILs were observed. (**F**) Sporadic PD-1+ TILs were observed. (**G**) High PD-1 expression was observed on cell menbranes of cancer cells. (**H**) Low PD-L1 expression. (**I**) H&E staining showed PC tissue with dense stroma. (**J**) H&E staining showed PC tissue with moderate stroma. Magnification, x200 (A–H); Magnification, x100 (I, J).

Immunohistochemistry (IHC) and haematoxylin-eosin (HE) staining results are summarized in Table 1 and Figure 1. At high magnification, MLH1 and MSH2 were distributed in the nucleus, while PD-1/PD-L1 was located on cell membranes. MLH1 was highly expressed in 63.8% (60/94) of PC samples (Figure 1A, 1B), while MSH2 was highly expressed in 75.5% (71/94) of PC samples (Figure 1C, 1D). High expression of PD-1 was observed in 46.8% (44/94) on tumor infiltrating lymphocytes (TILs) (Figure 1E, 1F). In contrast, PD-L1 was expressed on cell membranes of cancer cells with high expression in 28.7% (27/94) of PC tissues (Figure 1G, 1H). The density of tumor stroma was evaluated by haematoxylin-eosin staining. Dense stroma was observed in 55.3% (52/94) of cases (Figure 1I, 1J, Supplementary Figure S1).

**Table 1:** Results of MLH1, MSH2, PD-L1 and PD-1 IHC and HE staining

Maker	MLH1*n* (%)	MSH2*n* (%)	PD-L1*n* (%)	PD-1*n* (%)	Stroma^a^*n* (%)
Low score	34 (36.2%)	23 (24.5%)	67 (71.3%)	50 (53.2%)	42 (44.7%)
High score	60 (63.8%)	71 (75.5%)	27(28.7%)	44 (46.8%)	52 (55.3%)

^a^low score for stroma means moderate stroma, high score means dense stroma

### Correlations between MLH1/MSH2/PD-1/PD-L1 and clinicopathological characteristics

The relationships of the expression levels of MLH1, MSH2, PD-1 and PD-L1 with clinicopathological features of PC were evaluated. For MLH1 and MSH2, as summarized in Table 2, we did not observe any correlation between their expression levels and clinicopathological characteristics (Table 2). High expression of PD-L1 on cancer cell membranes correlated with lymph node metastasis (*P* = 0.033) and strongly correlated with poor-differentiation (*P* = 0.008), but did not correlate with patient's gender, age, tumor location, T stage, distant metastasis, vascular invasion and nervous invasion (*P* > 0.05) (Table 3). Meanwhile, high expression of PD-1 on cell membranes of TILs correlated with well-differentiation (*P* = 0.018) and strongly correlated with advanced T stage (*P* = 0.004), but did not correlate with patient's gender, age, tumor location, N stage, distant metastasis, vascular invasion and nervous invasion (*P* > 0.05) (Table 3). The relationships of the stroma density with clinicopathological features of PC were evaluated by haematoxylin-eosin staining. As summarized in Table 3, no correlation was observed between stroma density and patients’ clinicopathologic features.

**Table 2:** Correlation between the clinicopathologic characteristics and MLH1/MSH2 expression

	MLH1			MSH2		
	Low	High	*P* value	Low	High	*P* value
Age						
< 60 years	10 (34.5%)	19 (65.5%)	0.820^a^	9 (31.0%)	20 (69.0%)	0.323^a^
≥ 60 years	24 (36.9%)	41 (63.1%)		14 (21.5%)	51 (78.5%)	
Gender						
Male	24 (38.1%)	39 (61.9%)	0.580^a^	14 (22.2%)	49 (77.8%)	0.470^a^
Female	10 (32.3%)	21 (67.7%)		9 (29.0%)	22 (71.0%)	
Tumor site						
Head, neck	16 (33.3%)	32 (66.7%)	0.505^a^	14 (29.2%)	34 (70.8%)	0.306^a^
Body, tail	18 (40.0%)	27 (60.0%)		9 (20.0%)	36 (80.0%)	
T stage						
T1+T2	5 (25.0%)	15 (75.0%)	0.226^a^	3 (15.0%)	17 (85.0%)	0.382^a^
T3+T4	29 (39.7%)	44 (60.3%)		20 (27.4%)	53 (72.6%)	
N stage						
N0 (negative)	14 (35.0%)	26 (65.0%)	0.786^a^	7 (17.5%)	33 (82.5%)	0.160^a^
N1 (positive)	20 (37.7%)	33 (62.3%)		16 (30.2%)	37 (69.8%)	
Metastasis						
M0 (absent)	33 (37.1%)	56 (62.9%)	1.000^b^	21 (23.6%)	68 (76.4%)	0.255^b^
M1 (present)	1 (25.0%)	3 (75.0%)		2 (50.0%)	2 (50.0%)	
Differentiation						
G1 and G2	19 (31.1%)	42 (68.9%)	0.168^a^	11 (18.0%)	50 (82.0%)	0.094^a^
G3	15 (45.5%)	18 (54.5%)		11 (33.3%)	22 (66.7%)	
Vascular invasion						
No	32 (39.5%)	49 (60.5%)	0.199^a^	21 (25.9%)	60 (74.1%)	0.724^a^
Yes	2 (16.7%)	10 (83.3%)		2 (16.7%)	10 (83.3%)	
Nervous invasion						
No	11 (39.3%)	17 (60.7%)	0.720^a^	5 (17.9%)	23 (82.1%)	0.313^a^
Yes	23 (35.4%)	42 (64.6%)		18 (27.7%)	47 (72.3%)	

^a^Chi-square test; ^b^Fisher's exact test

**Table 3:** Correlation between the clinicopathologic characteristics and PD-1/PD-L1 expression

	PD-L1			PD-1			Stroma		
	Low	High	*P* value	Low	High	*P* value	Moderate	Dense	*P* value
Age									
< 60 years	19 (65.5%)	10(34.5%)	0.410^a^	16 (55.2%)	13 (44.8%)	0.797^a^	13 (44.8%)	16 (55.2)	0.985^a^
≥ 60 years	48 (73.8%)	17(22.2%)		34 (52.3%)	31 (47.7%)		29 (44.6%)	36 (55.4%)	
Gender									
Male	47 (74.6%)	16 (25.4%)	0.834^a^	37 (58.7%)	26 (41.3%)	0.125^a^	25 (39.7%)	38 (60.3%)	0.165^a^
Female	20 (64.5%)	11 (35.5%)		13 (41.9%)	18 (58.1%)		17 (54.8%)	14 (45.2%)	
Tumor site									
Head, neck	31 (64.6%)	17 (35.4%)	0.967^a^	24 (53.3%)	21 (46.7%)	0.936^a^	25 (52.1%)	23 (47.9%)	0.109^a^
Body, tail	35 (77.8%)	10 (22.2%)		26 (54.2%)	22 (45.8%)		16 (35.6%)	29 (64.4%)	
T stage									
T1+T2	14 (70.0%)	6 (30.0%)	0.914^a^	5 (25.0%)	15 (75.0%)	**0.004^a^**	12 (60.0%)	8 (40.0%)	0.106^a^
T3+T4	52 (71.2%)	21 (28.8%)		45 (61.6%)	28 (38.4%)		29 (39.7%)	44 (60.3%)	
N stage									
N0 (negative)	33 (82.5%)	7 (17.5%)	**0.033^a^**	21 (52.5%)	19 (47.5%)	0.832^a^	14 (35.0%)	26 (65.0%)	0.125^a^
N1 (positive)	33 (62.3%)	20 (37.7%)		29 (54.7%)	24 (45.3%)		27 (50.9%)	26 (49.1%)	
Metastasis									
M0 (absent)	63 (70.8%)	26 (29.2%)	0.999^b^	50 (56.2%)	39 (43.8%)	0.324^b^	38 (42.7%)	51 (57.3%)	0.317^a^
M1 (present)	3 (75.0%)	1 (25.0%)		1 (25.0%)	3 (75.0%)		3 (75%)	1 (25%)	
Differentiation									
G1 and G2	49 (80.3%)	12 (19.7%)	**0.008^a^**	27 (44.3%)	34 (55.7%)	**0.018^a^**	29 (47.5%)	32 (52.5%)	0.448^a^
G3	18 (54.5%)	15 (45.5%)		23 (69.7%)	10 (30.3%)		13 (39.4%)	20 (60.6%)	
Vascular invasion									
No	56 (69.1%)	25 (30.9%)	0.498^a^	43 (53.1%)	38 (46.9%)	0.734^a^	33 (40.7%)	48 (59.3)	0.091^a^
Yes	10 (83.3%)	2 (16.7%)		7 (58.3%)	5 (41.7%)		8 (66.7%)	4 (33.3%)	
Nervous invasion									
No	23 (82.1%)	5 (17.9%)	0.119^a^	14 (50.0%)	14 (50.0%)	0.633^a^	12 (42.9%)	16 (57.1%)	0.876^a^
Yes	43 (66.2%)	22 (33.8%)		36 (55.4%)	29 (44.6%)		29 (44.6%)	36 (55.4%)	

^a^Chi-square test; ^b^Fisher's exact test;

Significant values (*P* < 0.05) have been marked with bold.

### Prognostic impact of MLH1, MSH2, PD-1, PD-L1 and stroma density in PC

**Figure 2:**
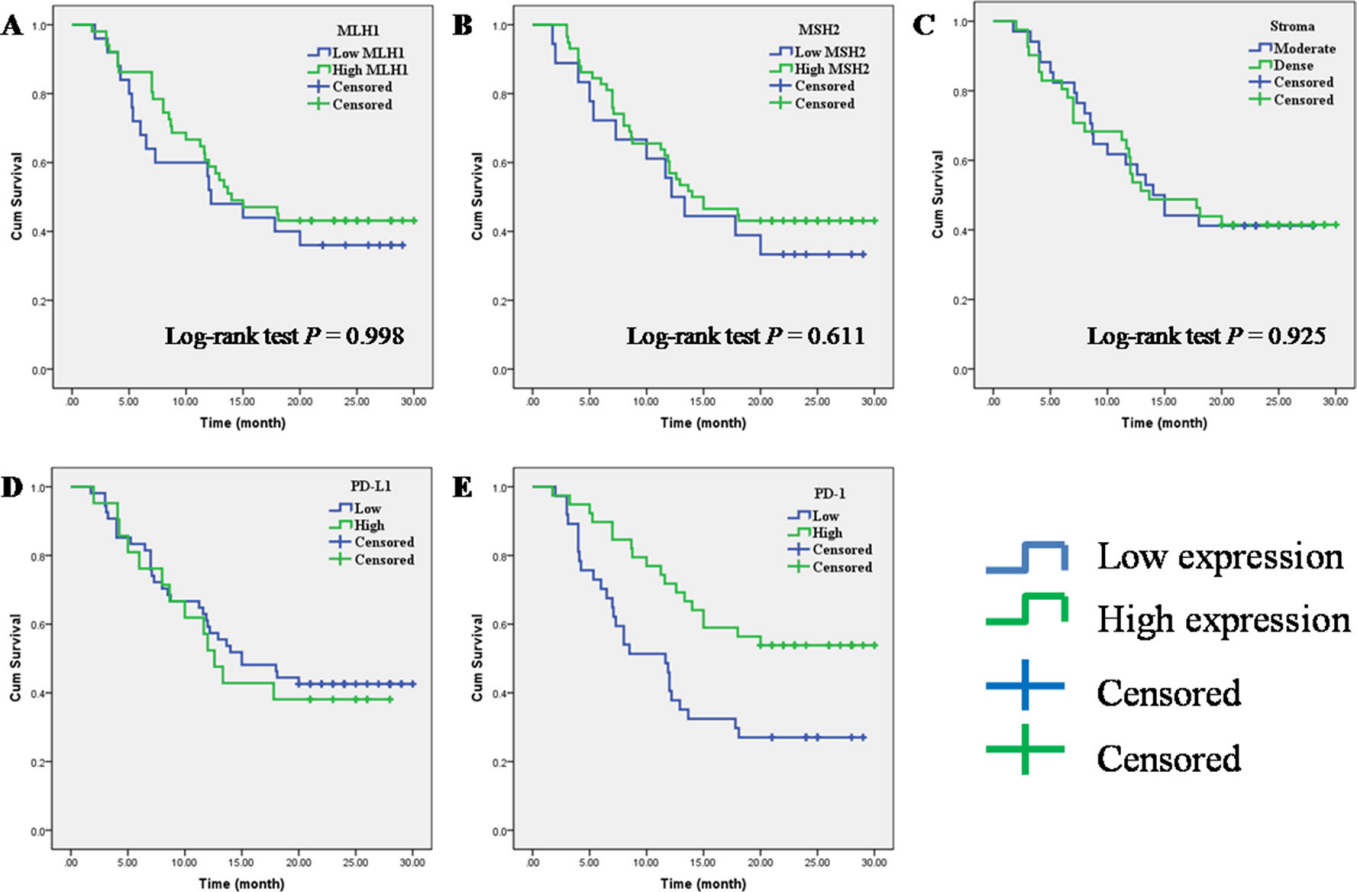
Prognostic impact of MLH1, MSH2, PD-1, PD-L1 and stroma density on overall survival in PC (**A**) MLH1; (**B**) MSH2; (**C**) stroma density; (**D**) PD-L1; (**E**) PD-1.

Kaplan-Meier analysis and log-rank test were used to evaluate the prognostic impact of MLH1, MSH2, PD-1, PD-L1, stroma density and clinicopathologic characteristics on patient survival. The log-rank test results showed that a high PD-1+ TILs expression had a significantly superior overall survival (OS) (log-rank test: *P* = 0.004) (Figure 2E), but we failed to observe any prognostic impact of stroma density and the expression of MLH1, MSH2 and PD-L1 (Figure 2A–2D). Then we used the Cox regression model to evaluate the correlation between prognostic factors and OS. As shown in Table 4, high PD-1+ TILs expression, well differentiation and no vascular invasion were associated with a significantly superior OS (*P* = 0.008, HR = 0.441, 95% CI 0.242–0.804), while poor differentiation and vascular invasion were associated with a poor OS (*P* = 0.026, HR = 1.963, 95% CI 1.084–3.553; *P* < 0.001, HR = 4.027, 95% CI 1.940–8.360). Finally, we conducted the multivariate analysis to investigate the prognostic factors together. Multivariate analysis showed that PD-1 TILs expression (*P* = 0.031; HR = 0.507; 95% CI 0.273–0.941), tumor differentiation (*P* = 0.006; HR = 2.504; 95% CI 1.307–4.798) and vascular invasion (*P* < 0.001; HR = 5.931; 95% CI 2.657–13.238) were independent prognostic factors for OS of PC patients.

**Table 4:** Univariate and multivariate Cox regression analysis of overall survival

	Univariate analysis			Multivariate analysis		
	HR	95% CI	*P* value	HR	95% CI	*P* value
Low MLH1	1					
High MLH1	0.798	0.433–1.470	0.470			
Low MSH2	1					
High MSH2	0.786	0406–1.523	0.476			
Low PD-1	1					
High PD-1	0.441	0.242–0.804	**0.008**	0.507	0.273–0.941	**0.031**
Low PD-1L	1					
High PD-1L	1.204	0.640–2.264	0.565			
Moderate Stroma	1					
Dense Stroma	0.972	0.541–1.747	0.925			
Age (< 60 years)	1					
Age (≥ 60 years)	0.765	0.415–1.409	0.390			
Gender (Male)	1					
Gender (Female)	0.896	0.482–1.666	0.728			
Tumor site (Head, neck)	1					
Tumor site (Body, tail)	1.486	0.824–2.680	0.188			
T stage (T1+T2)	1					
T stage (T3+T4)	1.992	0.889–4.467	0.094			
N stage (N0)	1					
N stage (N1)	1.212	0.670–2.191	0.525			
Metastasis (M0)	1					
Metastasis (M1)	0.584	0.141–2.413	0.457			
Differentiation (G1, G2)	1					
Differentiation (G3)	1.963	1.084–3.553	**0.026**	2.504	1.307–4.798	**0.006**
Vascular invasion (No)	1					
Vascular invasion (Yes)	4.027	1.940–8.360	**< 0.001**	5.931	2.657–13.238	**< 0.001**
Nervous invasion (No)	1					
Nervous invasion (Yes)	1.917	0.969–3.793	0.062			

HR: hazard ratio, 95% CI: 95% confidence interval.

Significant values (*P* < 0.05) have been marked with bold.

### Correlations of MMR gene expression, stroma density and PD-1/PD-L1 expression in PC tissues

**Figure 3:**
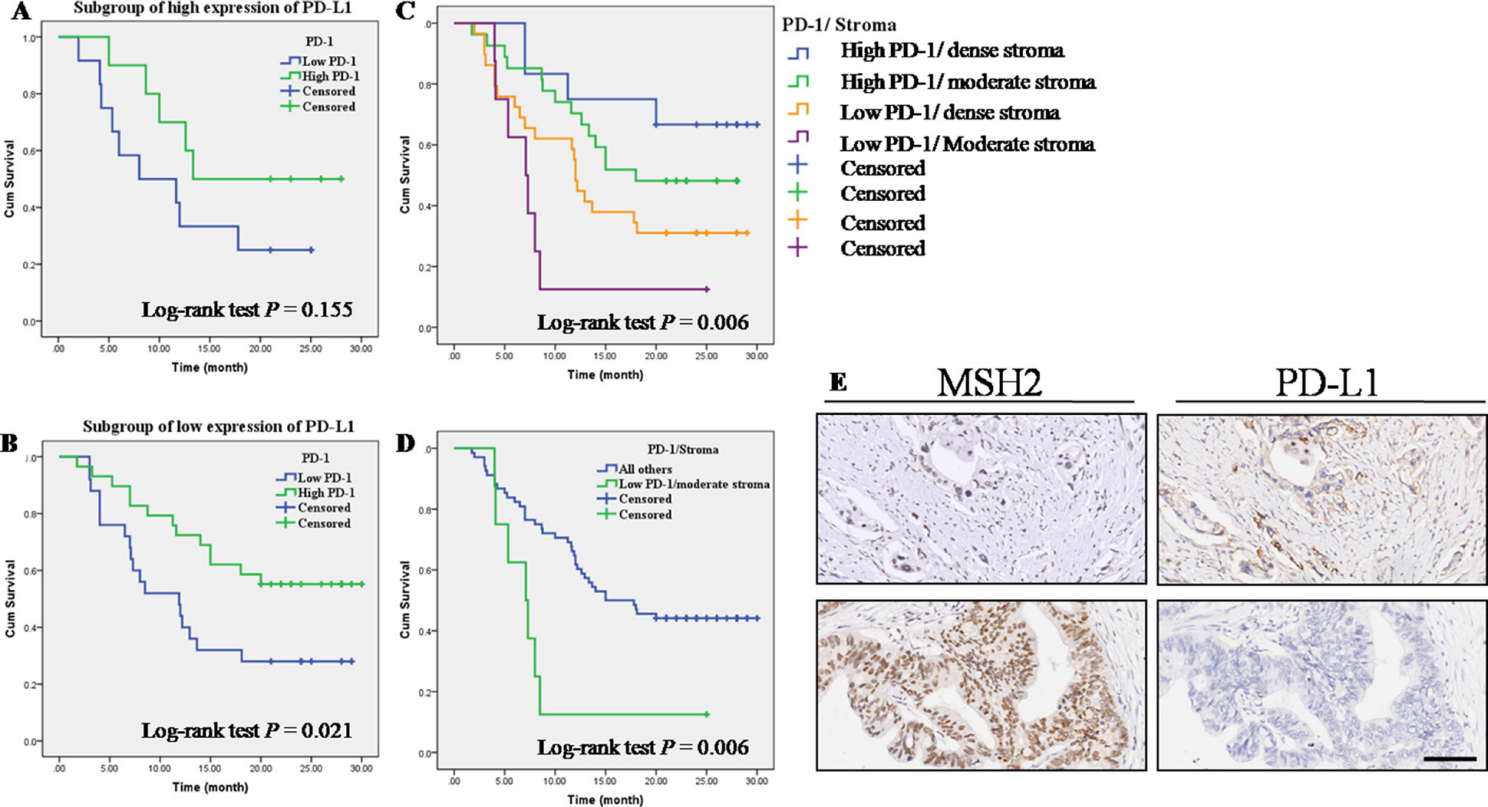
Correlations of MMR gene expression, stroma density and PD-1/PD-L1 expression in PC tissues (**A**) OS according to PD-1 expression in high PD-L1 expression subgroup. (**B**) OS according to PD-1 expression in low PD-L1 expression subgroup. (**C**) OS in four subgroups straitified by PD-1 expression and stroma density. (**D**) Low PD-1 expression and moderate stroma showed a worse outcome than the combination of other three group named “all others”. (**E**) Serial sections were used from the same PC tissue to depict an inverse correlation between MSH2 expression and PD-L1 expression. Top left and top right: low MSH2 expression vs. high PD-L1 expression; Lower left and lower right: high MSH2 expression vs. low PD-L1 expression.

As shown in Table 5, an inverse correlation was identified between low expression of MSH2 and high expression of PD-L1 (Spearman correlation coefficient r = −0.295, *P* = 0.004, Table 5). No correlations were found between PD-L1 expression and MLH1expression (*P* > 0.05, Table 5) and between PD-L1 expression and stroma density (*P* > 0.05, Table 5). Then, we used serial sections from the same PC tissue to depict the co-distribution of MSH2 and PD-L1 in PC tissue. As seen in Figure 3E, low MSH2 expression strongly correlated with high PD-L1 expression. For PD-1, as summarized in Table 6, we did not observe any correlation between PD-1 expression level and the expression of MMR gene (*P* > 0.05, Table 6). Also, PD-1 expression level was not associated with PD-L1 (*P* > 0.05, Table 6). Then, we further made subgroup analyses to evaluate the prognostic impact of PD-1 expression stratified by PD-L1. Kaplan-Meier analysis and log-rank test result showed that PD-1 expression level was associated with OS only at low PD-L1 subgroup (Figure 3A, 2B, P = 0.021). Moreover, high expression of PD-1 on cell membranes of TILs strongly correlated with moderate stroma (*P* < 0.001, Table 6). Previous studies indicated that a better survival was seen in surgically resected PC patients with higher levels of TILs within the tumor microenvironment and dense stroma made PC tissue poor-vascularized and can prevent T-cell from infiltrating (Supplementary Figure S3). To further investigate the association of OS with PD-1 expression and stroma density, we stratified the cases into four subgroups based on PD-1 expression and stroma density (Figure 3C). Kaplan-Meier analysis showed that the OS of four subgroups had significant differences (log-rank test: *P* = 0.006). Interesting, patients with high PD-1 expression and dense stroma had a better OS (median OS > 24 months), while patients with low PD-1 expression and moderate stroma showed a worst outcome (median OS = 7 months). Likewise, when the first three subgroups were combined into a single subgroup named “all others”, a similar result was found (log-rank test *P* = 0.006, Figure 3D).

**Table 5:** Association between PD-L1 and other markers

	PD-L1		Pearson *P* value	Spearman *P* value
	Low (*n*, %)	High (*n*, %)		
Low MLH1	22 (64.7%)	12 (35.3%)	R = –0.109	R = –0.109
High MLH1	45 (75.0%)	15 (25.0%)	*P* = 0.294	*P* = 0.294
Low MSH2	11 (47.8%)	12 (52.2%)	R = –0.295	R = –0.295
High MSH2	56 (78.9%)	15 (21.1%)	*P* = 0.004	*P* = 0.004
MSI	24 (60.0%)	16 (40.0%)	R = –0.214	R = –0.214
MSS	43 (79.6%)	11 (20.4%)	*P* = 0.038	*P* = 0.038
Moderate Stroma	27 (64.3%)	15 (35.7%)	R = –0.139	R = –0.139
Dense Stroma	40 (76.9%)	12 (23.1%)	*P* = 0.182	*P* = 0.182

Significant values (*P* < 0.05) have been marked with bold.

**Table 6:** Association between PD-1 and other markers

	PD-1		Pearson *P* value	Spearman *P* value
	Low (*n*, %)	High (*n*, %)		
Low MLH1	21 (61.8%)	13 (38.2%)	R = 0.129	R = 0.129
High MLH1	29 (48.3%)	31 (51.7%)	*P* = 0.214	*P* = 0.214
Low MSH2	13 (56.5%)	10 (43.5%)	R = 0.038	R = 0.038
High MSH2	37 (52.1%)	34 (47.9%)	*P* = 0.716	*P* = 0.716
MMR-D	23 (57.5%)	17 (42.5%)	R = 0.074	R = 0.074
MMR-P	27 (50.0%)	27 (50.0%)	*P* = 0.477	*P* = 0.477
Moderate Stroma	11 (26.2%)	31 (73.8%)	R = –0.486	R = –0.486
Dense Stroma	39 (75.0%)	13 (25.0%)	***P* < 0.001**	***P* < 0.001**
Low PD-L1	34 (50.7%)	33 (49.3%)	R = 0.077	R = 0.077
High PD-L1	16 (59.3%)	11 (40.7%)	*P* = 0.460	*P* = 0.460

Significant values (*P* < 0.05) have been marked with bold.

## DISCUSSION

PD-1/PD-L1 axis, as an immune checkpoint, has been widely studied in many different types of malignant. The expression of PD-1/PD-L1 was often used as a biomarker of immune checkpoint inhibitors [21–22]. Moreover, PD-1/PD-L1 antibody has been shown to be highly effective in gastrointestinal cancers as a single agent in patients with mismatch repair deficiencies. Deficient MMR results in the incorporation of mismatched nucleotides, with MMR-deficient colorectal cancer cells exhibiting 10–100 times the number of somatic mutations as those with proficient MMR [23–24]. Thus, high somatic mutation loads, which have the potential to create “non-self” immunogenic neoantigens, correlated with increased intratumoral effector T cell responses to the neoantigens Supplementary Tables S1, S2 [25]. Meanwhile, some studies indicated that MMR-deficient status was associated with higher expression of PD-L1 in gastric and colorectal cancer [26–28]. However, the relationship between MMR gene and PD-1/PD-L1 in pancreatic cancer remains largely unknown. Moreover, the correlations between PD-1/PD-L1 and prognosis are variants among different tumor types. Although Angela Diana et al. reported the expression of PD-1/PD-L1 and their prognostic value in pancreatic cancer [18]. However, the correlations of MMR-D/MMR-P and PD-1/PD-L1 and their clinical significant in pancreatic cancer were not fully understood, especially in East Asia.

Our study aimed at systematically analyzing the expression of MLH1, MSH2, PD-L1 and PD-1 in pancreatic cancer cells and tumor infiltrating cells, investigate their clinical significance and prognostic value. We found that high expression of PD-L1 on cancer cell membranes correlated with lymph node metastasis and strongly correlated with poor-differentiation, high expression of PD-1 on cell membranes of TILs correlated with well-differentiation and strongly correlated with advanced T stage, high PD-1 expression was associated with a significantly superior OS and was an independent prognostic factor. Then we found an inverse correlation between MSH2 expression and PD-L1 expression. In subgroup analyses, we observed that PD-1 expression level was associated with OS only at low PD-L1 expression subgroup. Finally, when we stratified the cases into four subgroups based on PD-1 expression and stroma density, we found that patients with high PD-1 expression and dense stroma had a better OS, while patients with low PD-1 expression and moderate stroma showed a worst outcome.

In our study, high expression of PD-1 on cell membranes of TILs strongly correlated with advanced T stage. Our observations are in line with most of previous studies in pancreatic cancer [18]. Also, our study reported that high expression of PD-L1 correlated with lymph node metastasis and strongly correlated with poor-differentiation. In previous study, Guo Y et al. made a meta-analysis to evaluate the prognostic and clinicopathological value of PD-L1 in breast cancer [29]. The result showed that positive/higher PD-L1 expression was significantly associated with positive lymph node metastasis and poor nuclear grade. Similar result was reported by Abbas M et al. in clear cell renal cell carcinoma [30]. The underline mechanisms remain unclear. Thus, part of the patients with metastatic lymph nodes, poorer nuclear grade may benefit from anti-PD-L1 therapy, which would improve their prognosis. PD-L1 expression was reported to be over expressed in MMR-deficient cancers, usually because MMR-deficient cancer creates high accumulated mutations which lead many tumor-specific neoantigens and is associated with high immune activities presumably aroused by mutated neoantigens. However, in present study, MSH2 was showed an inverse association with PD-L1 expression but not MLH1. These differences could be attributed to the several factors, such as the heterogeneity in population and different tumor type but also analysis method. In our study, high expression of PD-1 on cell membranes of TILs correlated with well-differentiation and a favorable clinical outcome in PC. Our results are in line with some studies in pancreatic cancer[18], but the mechanisms are currently unclear. Ashton et al. reported that antigen-specific immune activation following T-cell receptor stimulation leads to PD-1 upregulation on TILs [31]. Badoual et al. found higher expression of the immune activation markers HLA-DR and CD38 in PD-1+ TILs [32], so the presence of PD-1+ TILs could reflect an endogenous antitumor immune response that occurred upon activation of TILs. It could be an argument for this hypothesis that PD-1 expression level was associated with OS only at low PD-L1 expression subgroup. PC is characterised by an abundant desmoplastic stroma which is poor-vascularized and often prevents T-cell from infiltrating. The mechanisms include activated pancreatic stellate cells that drive apoptosis and sequestration of TILs, and the physical barrier imposed by dense collagen [33]. Analysis of PD-1+ TILs expression showed significantly higher infiltration in patients with moderate stroma density compared to patients with dense stroma and vice versa in our study. Moreover, patients with both low PD-1 expression and moderate stroma had a worse OS and vice versa. Intriguingly, Wang et al. reported stroma density constituted an independent prognostic marker in PC patients treated with adjuvant chemotherapy [8]. Some studies have reported higher TILs motility and migration in tumor areas with loose collagen [34]. Therefore, we speculated that low PD-1+ TILs in moderate stroma may reflect the lower TILs motility and migration and poor immune response of some patients. Obviously, these patients may have a worse outcome.

To our knowledge, the present study is the first study concerning PD-1/ PD-L1 and MMR statues analyses in East Asia population, which specifically investigated their clinical significant in PC. However, we would like to acknowledge the limitations of the study. Although we have analyzed a large cohort, the present study is a retrospective analysis and there is a potential for selection bias. And we did not include therapy responses in our studies which may affect the result of multivariate analysis.

In summary, we evaluated the correlation between clinicopathological characteristics and MMR, PD-1/PD-L1 expression, and found the prognosis value of PD-1 expression. In subgroup analyses, we observed that PD-1 expression level was associated with OS only at low PD-L1 expression subgroup. When we stratified the cases into four subgroups based on PD-1 expression and stroma density, we found that patients with high PD-1 expression and dense stroma had a better OS, while patients with low PD-1 expression and moderate stroma showed a worst outcome. Our result may provide more effective molecular markers for immunotherapeutic strategies of PC patients in clinical practice.

## MATERIALS AND METHODS

### Patients and treatment

This study was approved by the Ethics and Research Committees of Shanghai General Hospital, Shanghai Jiao Tong University School of Medicine, and was conducted in accordance with the Declaration of Helsinki Principles. The TMA used for this study includes 94 unselected, non-consecutive, primary, and sporadic PC treated between March 2012 and August 2014 in Pancreatic Cancer Center, Shanghai Jiao Tong University School of Medicine. Formalin-fixed, paraffin-embedded tissue blocks from resected PC were made. Tissue cylinders with a 2.0 mm diameter were punched from representative tissue areas. The histological types were confirmed by experienced pathologists. The TMAs contained well-documented clinicopathological information, including patients’ age, sex, location, tumor differentiation, T stage, lymph node metastasis, distant metastasis, nervous invasion, vascular invasion and follow-up data (ended in March, 2016). In total, 94 patients were included, 63 males and 31 females, with a median age of 62 years old (ranging from 31 to 78 years old). We got follow-up data of 77 patients in this cohort. The overall survival time ranged from 1.75 to 30.00 months, with a median of 14 months. Detailed information can be found in Table 7.

**Table 7:** Detailed clinical information of patients in our study

	Categories	Numbers
Overall survival		14 months, range (1.75–30.00)
Age		62 years, range (31–78)
Gender	Male	63
	Female	31
Tumor site	Head, neck	48
	Body, tail	45
T stage	T1+T2	20
	T3+T4	73
N stage	N0 (negative)	40
	N1 (positive)	53
Metastasis	M0 (absent)	89
	M1 (present)	4
Differentiation	G1	10
	G2	51
	G3	33
Vascular invasion	No	81
	Yes	12
Nervous invasion	No	28
	Yes	65

### Immunohistochemical staining

Immunohistochemistry was performed based on the standard streptavidin-peroxidase (S-P) method (Zymed, San Francisco, CA). Briefly, the tissue microarrays were dewaxed and dehydrated in a xylene and alcohol bath solution. Heat mediated antigen retrieval was performed using Tris/EDTA buffer pH 9.0. After that, endogenous peroxidase activity was then blocked with 0.3% hydrogen peroxide for 10 mins. The slides were cooled to room temperature and blocked by incubating with normal goat serum at room temperature for 1h and were subsequently incubated overnight at 4°C with primary antibodies as follows: PD-1 (dilution1:50, Abcam), PD-L1 (dilution 1:500, Abcam), MLH1 (dilution1:500, Abcam), MSH2 (dilution 1:500, CST) (Supplementary Figure S2).

The sections were incubated with biotinylated secondary antibodies (Zymed, San Francisco, CA) for 30 min at room temperature, followed by incubation with streptavidin horseradish peroxidase complex. Finally, sections were visualized by 3, 3′-diaminobenzidine staining. Meanwhile, slides were stained with haematoxylin and eosin (H&E) as described before.

### Scoring of immunohistochemistry

Immunostaining signals were evaluated independently by two experienced pathologists without access to the patients’ clinical and pathological features. MLH1, MSH2 and PD-L1 expression were scored according to staining intensity and the percentage of positive cells as previously described [35]. The staining intensity was scored as 0 (negative), 1 (weak), 2 (medium) or 3 (strong). The percentage of positive cells was scored as follows: < 5% (0), 5%–25% (1), 25%–50% (2), 50%–75% (3) and 75%–100% (4) according to the percentages of the positive staining areas in relation to the whole carcinoma area. Comprehensive score = staining percentage × intensity.

Samples with a final staining score of < 6 were classified as low expression, while those with score of ≥ 6 were considered to be high expression. The extent of TILs was assessed in HE stained TMA. The expression of PD-1 in TILs by IHC method was evaluated by measuring cell density as previously reported. Scoring was as follows: (1) absent cells; (2) < 25% cell density; (3) 25–50% cell density; (4) >50% cell density. Samples with a score of 1 or 2 were considered negative (low expression) and those with a score of 3 or 4 were considered positive (high expression). Stromal density based on HE staining, and classified as moderate or dense as previously described. The stromal density was evaluated on H&E-stained TMAs. Quality was defined as moderate or strong on the basis of its morphologic appearance. Moderate stroma had a paucicellular matrix of loosely packed connective tissue fibres with occasional oedematous appearance. Cases with dense stroma showed a densely packed network of fibres with intense staining. Thus, dense stroma presents mature collagen fibres packed into multilayers with intense staining and others were considered to be “moderate” [7–8, 35].

### Statistical analysis

All statistical analyses were carried out by the SPSS 16.0 software. The χ^2^ test and Fisher's exact test were used to analyze the correlations between categorical variables. Overall survival (OS) was defined as the interval from date of diagnosis until death from any cause. Data were censored for living patients and patients lost between follow-ups. The OS was estimated using the Kaplan-Meier method and compared using the log-rank test. Significant variables were further analyzed by multivariate analysis to test for independent prognosis. Bivariate correlations between variable factors were calculated by Spearman rank correlation coefficients. *P*-values < 0.05 were considered statistically significant.

## SUPPLEMENTARY MATERIALS


