# Supplementary material for: Prognostic value and clinicopathological features of PD-1/PD-L1 expression with mismatch repair status and desmoplastic stroma in Chinese patients with pancreatic cancer
Source: Oncotarget. 2016 Dec 21;8(6):9354–65. doi: 10.18632/oncotarget.14069 (PMC5354736; doi:10.18632/oncotarget.14069)
Supplement: Supplementary file 1 [file oncotarget-08-9354-s001.pdf]

Prognostic value and clinicopathological features of PD-1/PD-L1 expression with mismatch repair status and desmoplastic stroma in Chinese patients with pancreatic cancer

Supplementary Materials

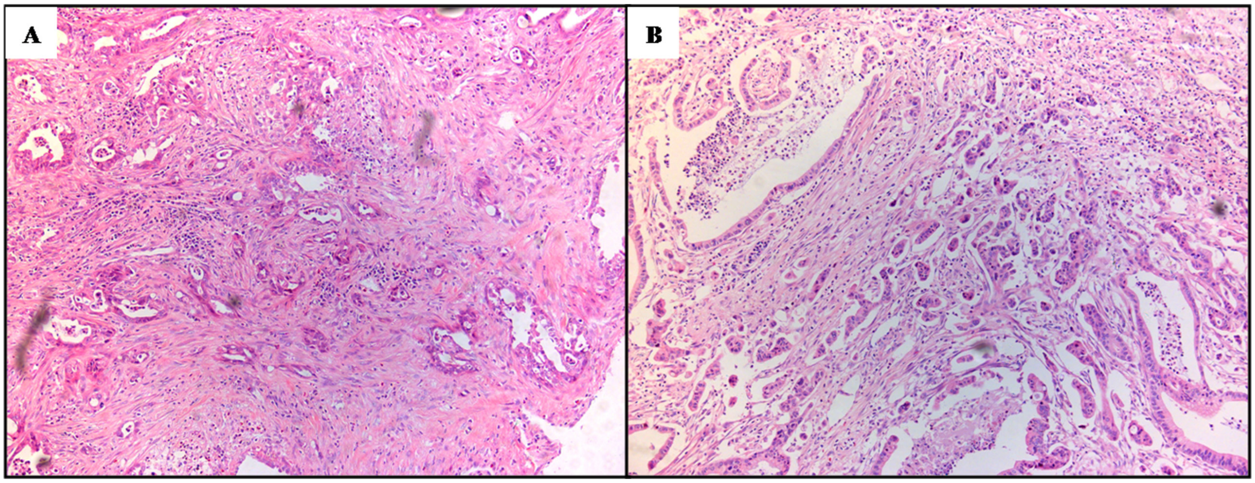

Supplementary Figure S1: H&E staining of stroma density. (**A**) H&E staining showed PC tissue with dense stroma. (**B**) H&E staining showed PC tissue with moderate stroma. Magnification, × 100.

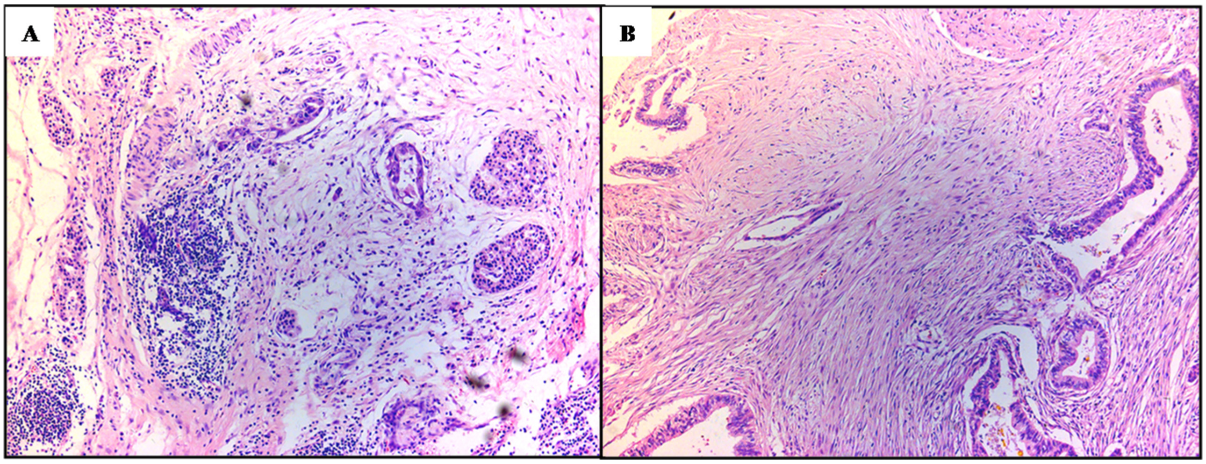

Supplementary Figure S2: H&E staining of T lymphocytes (TLCs) density. (**A**) high density of TLCs: abundant occurrence of T-cells; (**B**) low density of TLCs: sporadic T-cells. Magnification, × 100.

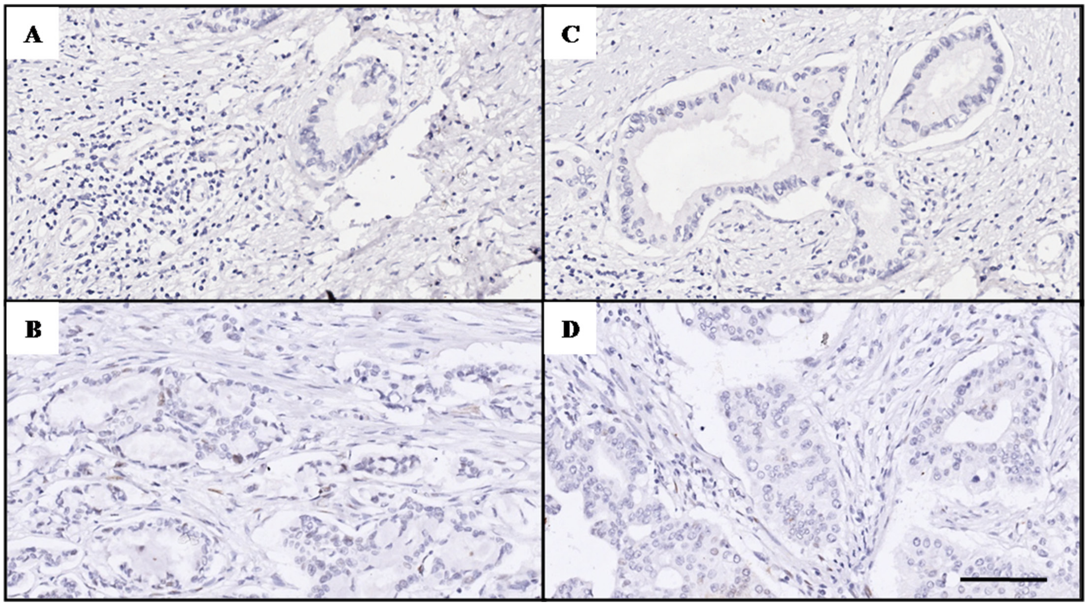

Supplementary Figure S3: Immunohistochemical staining of negative control for PD-1. (**A**) PD-L1 (**B**) MLH1 (**C**) and MSH2 (**D**) in PC tissues. Scale bar, 50 μm.

Supplementary Table S1: Association between TLC and other markers

	TLC		Pearson	Spearman
	**Low (*n*, %)**	**High (*n*, %)**	***P* value**	***P* value**
Low MLH1	17 (50.0%)	17 (50.0%)	R = –0.064	R = –0.064
High MLH1	34 (56.7%)	26 (25.0%)	P = 0.538	P = 0.538
Low MSH2	8 (34.8%)	15 (65.2%)	R = –0.222	R = –0.222
High MSH2	43 (60.6%)	28 (39.4%)	**P = 0.031**	**P = 0.031**
Low PD-1	39 (78.0%)	11 (22.0%)	R = 0.508	R = 0.508
High PD-1	12 (27.3%)	32 (72.7%)	**P < 0.001**	**P < 0.001**
Low PD-L1	40 (59.7%)	27 (40.3%)	R = 0.172	R = 0.172
High PD-L1	11 (40.7%)	16 (59.3%)	P = 0.097	P = 0.097

Significant values (P < 0.05) have been marked with bold.

Supplementary Table S2: Correlation between the clinicopathologic characteristics and TLC

	TLC		
	**Low dense**	**High dense**	***P* value**
Age			
< 60 years	15 (51.7%)	14 (48.3%)	0.742[a]
≥ 60 years	36 (55.4%)	29 (44.6%)	
Gender			
Male	37 (58.7%)	26 (41.3%)	0.068[a]
Female	12 (38.7%)	19 (61.3%)	
Tumor site			
Head, neck	24 (50.0%)	24 (50.0%)	0.333[a]
Body, tail	27 (60.0%)	18 (40.0%)	
T stage			
T1+T2	9 (45.0%)	11 (55.0%)	0.318[a]
T3+T4	42 (57.5%)	31 (42.5%)	
N stage			
N0 (negative)	22 (55.0%)	18 (45.0%)	0.978[a]
N1 (positive)	29 (54.7%)	24 (45.3%)	
Metastasis			
M0 (absent)	50 (56.2%)	39 (43.8%)	0.220[b]
M1 (present)	1 (25.0%)	3 (75.0%)	
Differentiation			
G1 and G2	33 (54.1%)	28 (45.9%)	0.967[a]
G3	18 (54.5%)	15 (45.5%)	
Vascular invasion			
No	44 (54.3%)	37 (45.7%)	0.794[a]
Yes	7 (58.3%)	5 (41.7%)	
Nervous invasion			
No	15 (53.6%)	13 (47.4%)	0.872[a]
Yes	36 (55.4%)	29 (44.6%)	

[a]Chi-square test;[b]Fisher's exact test.
